# Tanshinone IIA Inhibits Epithelial-to-Mesenchymal Transition Through Hindering β-Arrestin1 Mediated β-Catenin Signaling Pathway in Colorectal Cancer

**DOI:** 10.3389/fphar.2020.586616

**Published:** 2020-10-29

**Authors:** Qing Song, Liu Yang, Zhifen Han, Xinnan Wu, Ruixiao Li, Lihong Zhou, Ningning Liu, Hua Sui, Jianfeng Cai, Yan Wang, Qing Ji, Qi Li

**Affiliations:** ^1^ Department of Medical Oncology and Cancer Institute of Integrative Medicine, Shuguang Hospital, Shanghai University of Traditional Chinese Medicine, Shanghai, China; ^2^ Academy of Integrative Medicine, Shanghai University of Traditional Chinese Medicine, Shanghai, China; ^3^ Department of Medical Oncology, Suzhou TCM Hospital Affiliated to Nanjing University of Chinese Medicine, Suzhou, China; ^4^ Department of Oncology, Baoshan Branch, Shuguang Hospital Affiliated to Shanghai University of Traditional Chinese Medicine, Shanghai, China; ^5^ Department of Chemistry, University of South Florida, Tampa, FL, United States

**Keywords:** tanshinone IIA, colorectal cancer, epithelial-to-mesenchymal transition, β-arrestin1, β-catenin signaling pathway

## Abstract

Tanshinone IIA (Tan IIA) is a major active ingredient extracted from Salvia miltiorrhiza, which has been proved to be able to inhibit metastasis of various cancers including colorectal cancer (CRC). However, the mechanisms of anti-metastatic effect of Tan IIA on CRC are not well explored. A number of studies indicate that epithelial-to-mesenchymal transition (EMT) plays an important role in CRC metastasis, and our previous studies demonstrate that β-arrestin1could regulate EMT in CRC partly through β-catenin signaling pathway. In this work, we investigate whether Tan IIA could regulate EMT in CRC through β-arrestin1-mediated β-catenin signaling pathway both *in vivo* and *in vitro*. Our results showed that Tan IIA inhibited lung metastases of CRC cells *in vivo* and extended the survival time of mice with CRC. *In vitro*, Tan IIA increased the expression of E-cadherin, decreased the expression of Snail, N-cadherin and Vimentin, thus suppressed EMT and the migratory ability of CRC cells. Further study found that the mechanism of action of Tan IIA in regulating EMT and metastasis is associated with the suppression of β-arrestin1 expression, resulting in the increase of GSK-3β expression, reduction of β-catenin nuclear localization, thereby decreased the activity of β-catenin signaling pathway. Our data revealed a new mechanism of Tan IIA on the suppression of EMT and metastasis in CRC via β-arrestin1-mediated β-catenin signaling pathway and provided support for using Tan IIA as anti-metastatic agents in CRC.

## Introduction

Colorectal cancer (CRC) is the third most common cancer and the second cause of cancer-associated death in the world, consisting of over 1.7 million new cases and 861,663 deaths per year (Bray et al., 2018). CRC has a high mortality rate because more than half of the patients are diagnosed at advanced stages or recurrence and metastasis after tumor resection (Vanhara and Soucek, 2013). Therefore, exploring the molecular mechanism of CRC metastasis, preventing recurrence and metastasis of CRC, and improving the survival of CRC patients have become an urgent need.

Epithelial-to-mesenchymal transition (EMT) plays a key role in CRC metastasis (Li et al., 2017). Generally, epithelial cells lose cell polarity and the ability of cell adhesion, penetrate the basement membrane and enter the circulatory system, then initiate and promote the invasion and metastasis of cancer cells (Vu and Datta, 2017). During EMT, down-regulation of E-cadherin leads to the loss of cell adhesion, upregulation of Vimentin and N-cadherin causes the reorganization of actin cytoskeleton and promotes cell motility (Guarino et al., 2009). Upregulation of transcription factors such as Snail, Slug and Twist induces EMT (Goossens et al., 2017). In addition, TGF-β/SMADs (Mishra et al., 2017), PI3K/Akt (Tan et al., 2017) and other signaling pathways participate in regulating EMT. GSK/β-catenin signaling plays an important role in tumor EMT, invasion and metastasis. Inactivation of GSK-3β phosphorylation facilitates the nuclear localization of β-catenin, leading to the activation of GSK/β-catenin signaling pathway, increasing the expression of snail, ZEB1 and Twist, thereby promoting tumor EMT and metastasis (Wang et al., 2018; Zhang et al., 2018a). In our previous study, we found that β-arrestin1 had higher expression in lung metastases of CRC than in primary tumor. Moreover, β-arrestin1 could promote EMT and metastasis through repressing the expression of GSK-3β, inhibiting the degradation of β-catenin, thereby activating β-catenin signaling pathway (Supplementary Material for review).

Tanshinone is an ether or ethanol extract from the root of Salvia miltiorrhiza, and Tan IIA is its major active constituent. Several studies have showed the pharmacological effects of Tan IIA on cell apoptosis, metastasis, drug resistance, and angiogenesis in different cancers (Sui et al., 2017; Qian et al., 2018; Zhang et al., 2018b). Zhou et al. found that Tan IIA Tan IIA not only decreased HIF-1α expression and inhibited the secretion level of vascular endothelial growth factor and basic fibroblast growth factor, but also efficiently decreased proliferation, tube formation and metastasis of HUVECs (Zhou et al., 2020). However, the mechanism of action of Tan IIA on EMT and tumor metastasis in CRC is not well elucidated. In this study, we explore the effect and mechanism of Tan IIA on EMT and metastasis of CRC both *in vivo* and *in vitro*. This study will provide a rational for application of Tan IIA for the treatment of CRC.

## Materials and Methods

### Cell Culture and Reagents

Human colorectal cancer cell line HCT-116 (ATCC, United States) was cultured in 1640 medium and LoVo (ATCC, United States) was cultured in F-12K medium, containing 10% fetal bovine serum (FBS), 100 U/ml penicillin, and 100 mg/ml streptomycin at 37°C in a 5% CO_2_ incubator. Rabbit monoclonal antibodies against human E-cadherin, N-cadherin, Vimentin, Snail, β-arrestin1, GSK-3β, β-catenin, c-Myc, and CyclinD1 were purchased from Cell Signaling Technology (United States). Rabbit monoclonal antibodies against human MMP2 and MMP9 were purchased from Santa Cruz Biotechnology (United States). Mouse monoclonal antibody against human GAPDH was purchased from Proteintech (China). Tanshinone IIA (S2365) with 99% purity (HPLC) was purchased from Selleck (Houston, TX, United States).

### Cell Viability Assay

Cell Counting Kit-8 (CCK-8) was used to detect cell proliferation. Cells at 1 × 10^4^ cells/well were seeded into 96-well plates. When the cell density reached 60%, different concentrations of Tan IIA were added into the wells and incubated for 24, 48, and 72 h. Then the medium containing CCK-8 reagent was added into the cells and incubated for 4 h. The light absorption was measured at 450 nm with a microplate reader (Biorad, United States). All experiments were carried out in six wells, and each experiment was repeated at least three times.

### Western Blot

In brief, all cells were lyzed with RIPA Lysis buffer, and the extracted protein was quantified by BCA protein assay (Beyotime Biotechnology, Shanghai, China). Approximately 50 μg of proteins were added into the 10% SDS-PAGE gels for electrophoresis and then transferred onto a polyvinylidene difluoride (PVDF) membrane. After that, PVDF was blocked with 5% BSA, and incubated with the primary antibodies followed by the HRP-conjugated secondary antibodies. The results were examined with enhanced chemiluminescence (ECL, Millipore, CA, United States), and the target bands were analyzed using the Scion Imaging application (Scion Corporation).

### Immunofluorescence Microscopy

HCT-116 and LoVo cells were fixed with 4% paraformaldehyde for 30 min at room temperature, permeabilized with Triton X-100 (0.5%) for 15 min, then blocked with 5% BSA solution for 1 h. The cells were incubated with primary antibodies for 1 h, followed by secondary antibodies for 1 h at room temperature. After incubation, nucleus was labeled with DAPI for 5 min. Finally, cells were examined with a DMI3000B inverted microscope (Leica, Germany).

### Transwell Assay for Cell Migration

HCT-116 and LoVo cells were inoculated into the upper chamber of transwell plates and cultured in 600 μl 1640 or F12K medium with 10 μg/ml fibronectin. Medium containing 15% FBS was added in the lower chamber of the transwell plate. Then different concentrations of Tan IIA were added into the upper chamber and incubated for 48 h. Migrated cells were detected by crystal violet staining and observed using the DMI3000B inverted microscope (Leica, Germany). Five random views were selected to count the migrated cells.

### Wound-Healing Assay

HCT-116 or LoVo cells were seeded into 6-well plates for 24 h, then an artificial scratch wound was created using a 20 μl pipette tip and detached cells were removed by washing with PBS three times. After 48 h incubation, cell migration was photographed using the inverted microscope and evaluated by measuring the difference in wound width.

### Animal Model

Male BALB/c nude mice (5 weeks old) were purchased from the Department of Experimental Animals of Shanghai University of Traditional Chinese Medicine (Shanghai, China, license No. SCXK 2018-0006) and maintained in the specific pathogen-free condition for 1 week. The mice were injected with 2 × 10^6^ Luc-labeled HCT-116 cells through the tail vein. Two weeks later, the mice were randomly divided into five groups, eight in each group. The mice were treated with different concentrations of Tan IIA (0.5, 1, and 2 mg/kg) for 4 weeks by tail vein injection. For bioluminescence imaging, mice were anesthetized with 1% pentobarbital sodium (200 μl/per), and D-luciferin (15 mg/ml) was injected intraperitoneally. The data were captured by using an IVIS Lumina system (Caliper, United States). After that, the lungs were excised, fixed in 4% paraformaldehyde, and paraffin-embedded. The paraffin-embedded lung tissues were cut into 5 μm sections. All the lung sections were stained with hematoxylin-eosin (HE). E-cadherin, Vimentin, Snail, and β-arrestin1 were detected with immunohistochemistry (IHC). The animal experiments were performed under the approval of the animal ethics committee of Shuguang Hospital, Shanghai University of Traditional Chinese Medicine.

### Statistical Analysis

All the data were presented as means ± SD of at least three independent experiments and analyzed using SPSS22 Software. The mean values of two groups were compared by Student’s t test. *p* < 0.05 was considered as statistically significant.

## Results

### Tan IIA Inhibited Lung Metastasis of Colorectal Cancer *In Vivo*

**FIGURE 1:**
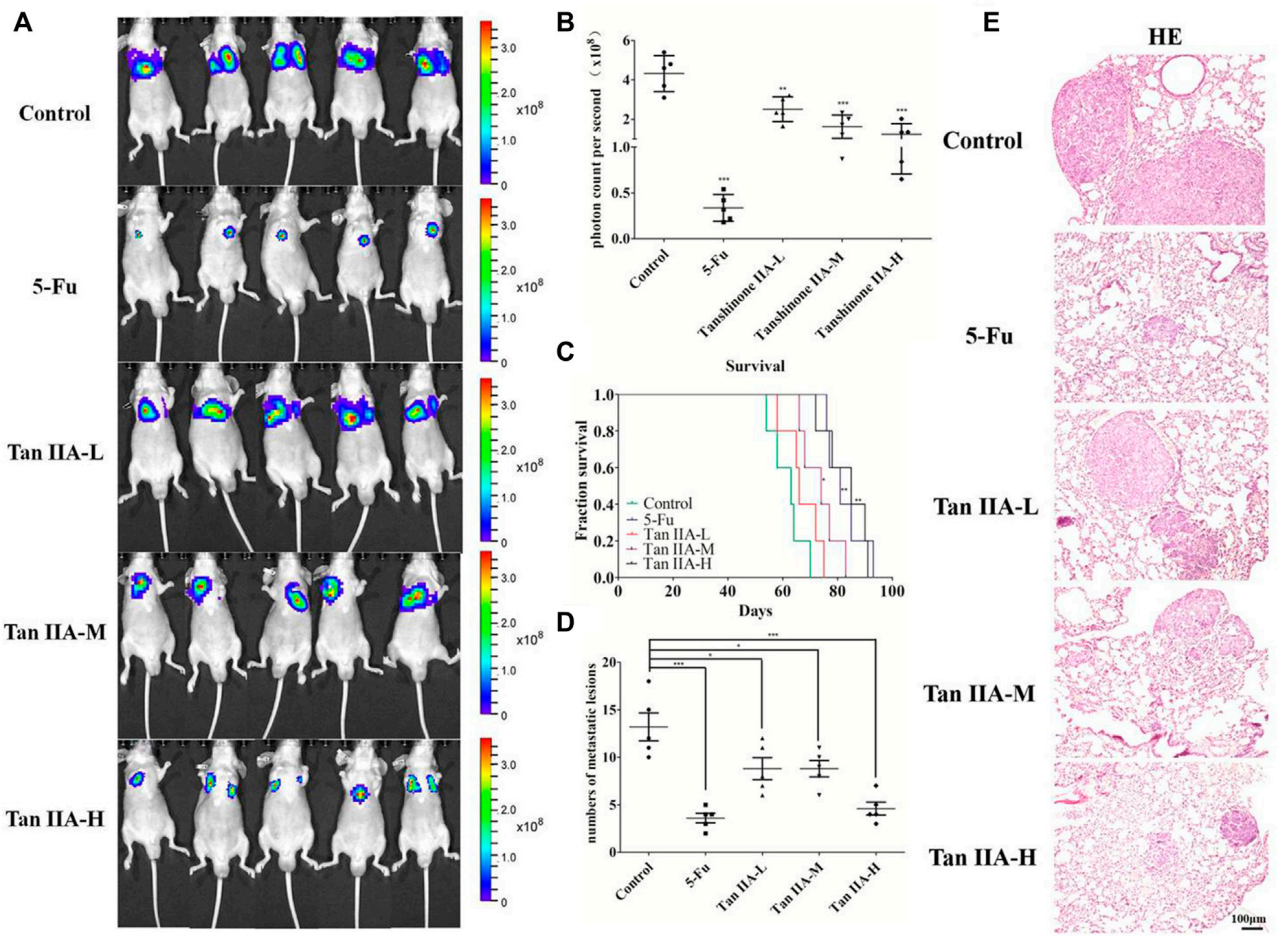
Tan IIA inhibited the metastasis of colorectal cancer *in vivo*. **(A,B)** Each group of mice was injected with HCT-116/luc cells through the tail vein. After treatment with Tan IIA at concentrations of 0.5, 1, and 2 mg/kg for 4 weeks, luciferase imaging data was collected by IVIS Lumina system. ***p < 0.001, compared with control group. **(C)** The survival of tumor-bearing mice were evaluated, *p < 0.05; **p < 0.01; ***p < 0.001, compared with control group. **(D,E)** The lung tumors were excised, hemaoxylin-eosin (H&E) staining was performed and the number of metastatic lesions were counted, *p < 0.05; ***p < 0.001, compared with control group.

Tan IIA is a major active ingredient extracted from Salvia miltiorrhiza, which has been proved to be able to inhibit metastasis of various cancers. In the present study, to assess the effect of Tan IIA on CRC metastasis *in vivo*, we established a CRC lung metastasis mouse model by tail vein injection of CRC cells. The mice were treated with different concentrations of Tan IIA. *In vivo* imaging results indicated that HCT-116/luc cells migrated to lung tissues after tail injection, and Tan IIA at concentrations of 0.5, 1, and 2 mg/kg (Sui et al., 2017) inhibited the metastatic ability of HCT-116/luc cells in a concentration-dependent manner. After treatment with Tan IIA for 4 weeks, the size of fluorescent tumor is significantly reduced in treated mice compared to control group **(** Figures 1A,B **)**. Also, Tan IIA improved the survival of mice. The survival time of mice (the life prolongation rates) of low, medium and high dose of Tan IIA in tumor-bearing mice increased 8.73%, 19.09% and 34.63%, respectively compared to control group **(** Figure 1C **)**. Furthermore, the numbers of lung metastases in Tan IIA treated mice were significantly fewer than control group. The number of lung metastases was the least in the high-dose Tan IIA treated group **(** Figure 1D **)**. HE staining was used to observe lung metastases in nude mice, which was consistent with *in vivo* imaging results **(** Figure 1E **)**.

### Tan IIA Inhibited the Epithelial-to-Mesenchymal Transition of Colorectal Cancer Cells

**FIGURE 2:**
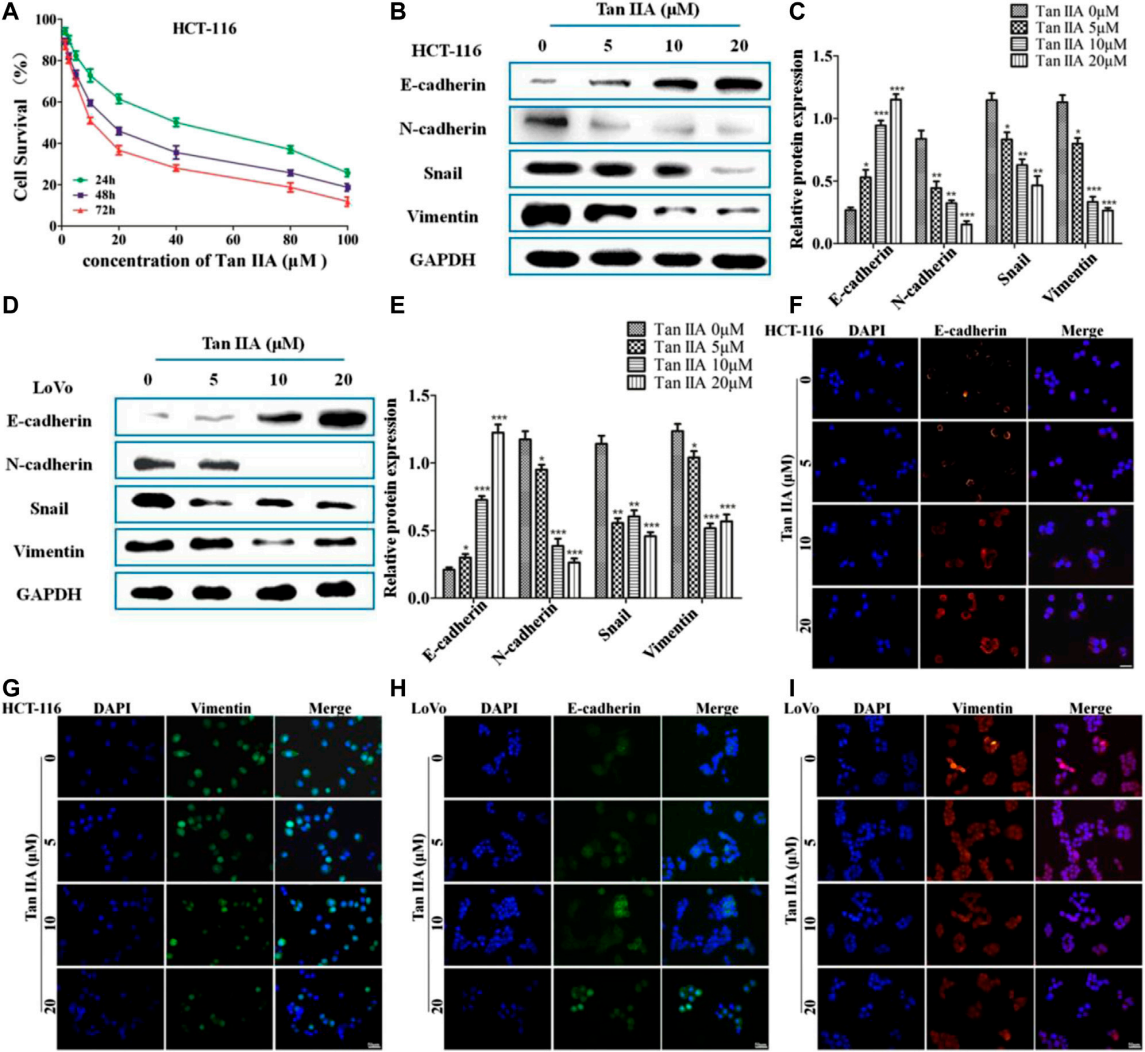
Tan IIA inhibited EMT in colorectal cancer cells. **(A)** CCK-8 assay on the cellular inhibition of Tan IIA (0, 1.25, 2.5, 5, 10, 20, 40, 80, and 100 μM) in HCT116 cells for 24, 48, and 72 h. The experiment was performed three times with similar results. **(B–E)** HCT-116 cells and LoVo cells were treated with Tan IIA at 0, 5, 10, and 20 μM, Western blot was used to detect the expression of E-cadherin, N-cadherin, Snail, and Vimentin. The data were presented as the mean ± SD from at least three experiments. *p < 0.05; **p < 0.01; ***p < 0.001, compared with group without treatment of Tan IIA. **(F–I)** The effect of Tan IIA on epithelial biomarker E-cadherin and mesenchymal biomarker Vimentin detected by immunofluorescent staining. The data were performed at least three experiments.

The above studies indicated that Tan IIA inhibited lung metastasis of colorectal cancer *in vivo*. We explored the potential anti-metastatic mechanism of Tan IIA *in vitro*. EMT is characterized with the loss of adherent junctions (AJs). E-cadherin, N-cadherin, Vimentin, and Snail are the protein molecules responsible for EMT (Przybyla et al., 2016). We examined anticancer activity of Tan IIA on HCT-116 and LoVo cells with different concentrations. The data showed that Tan IIA inhibited proliferation of HCT-116 cells **(** Figure 2A **)** and LoVo cells (Supplementary Figure S1) in a concentration-and time-dependent manner. The IC50 value of Tan IIA was 17.48 μM at 48 h in HCT-116 cells. We used the concentration of 0, 5, 10, and 20 μM and 48 h as the dosages and treatment time in the following experiments.

In order to estimate the effect of Tan IIA on EMT, we examined the expression of E-cadherin, N-cadherin, Vimentin, and Snail by Western blot and immunofluorescence (Jeong et al., 2018). The results revealed that, treatment with Tan IIA reduced the expression of N-cadherin, Vimentin, and Snail, and increased the expression of E-cadherin in a concentration-dependent manner **(** Figures 2B–E **)**. Immunofluorescence images demonstrated that, the expression of E-cadherin on the membrane increased, but the expression of Vimentin in the cytoplasm decreased after cells treated with Tan IIA (Figures 2F–I), which was similar to the results of Western blot.

### Tan IIA Inhibited the Migration of Colorectal Cancer Cells

**FIGURE 3:**
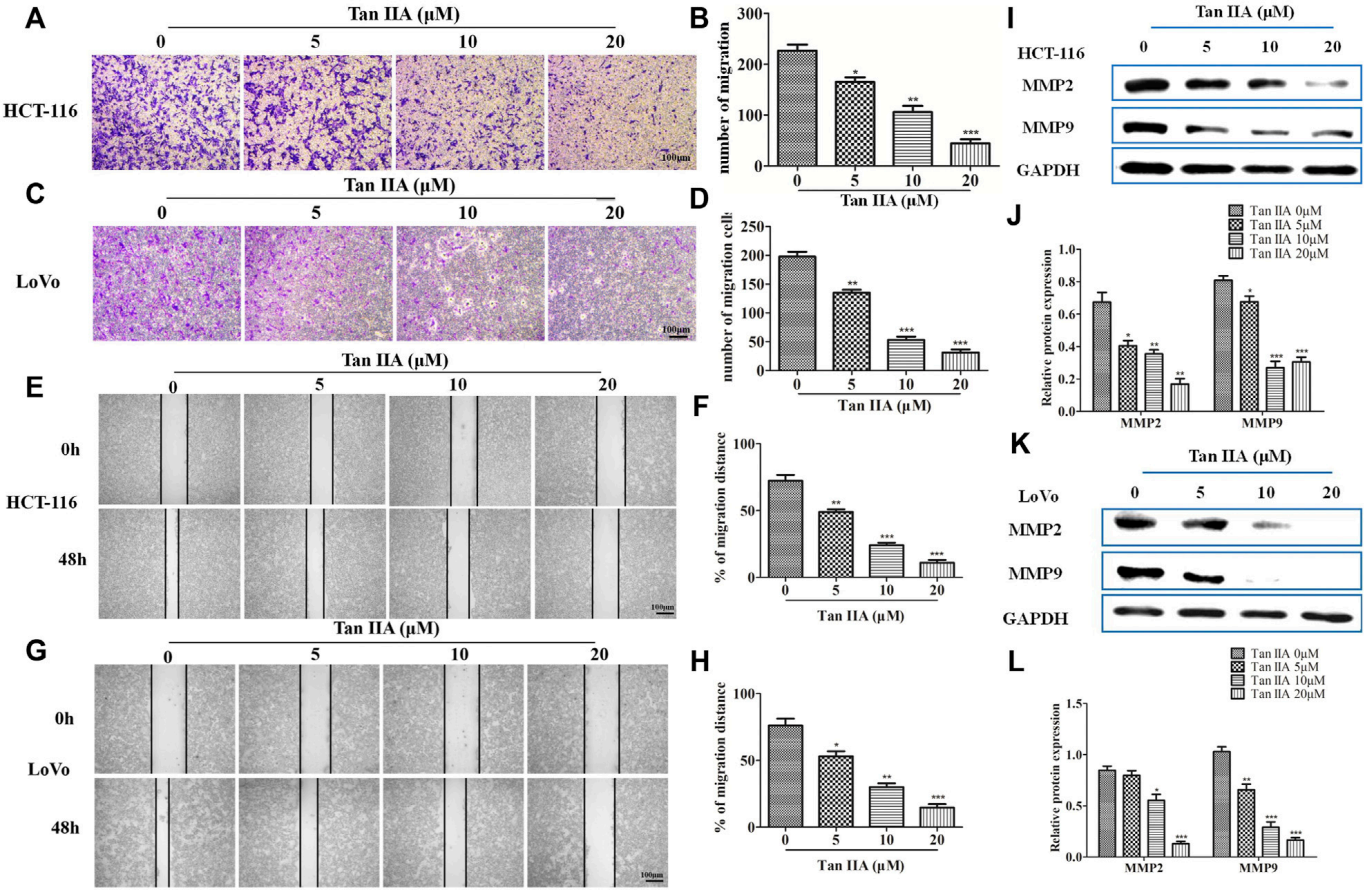
Tan IIA inhibited the migration of CRC cells. **(A-D)** HCT-116 and LoVo cells were treated with different concentration of Tan IIA, and transwell assay was used to detect the migratory cells counted from five random microscopic fields. The experiment was performed three times with similar results. *p < 0.05; **p < 0.01; **p < 0.01, compared with group without treatment of Tan IIA. **(E–H)** HCT-116 and LoVo cells treated with or without Tan IIA for 48 h, the wound-healing assay data were shown. The black lines were used to mark the borders of the scratches, *p < 0.05; **p < 0.01; **p < 0.01, compared with group without treatment of Tan IIA. The data were presented as the mean ± SD from at least three experiments. **(I–L)** The expression of MMP-2 and MMP-9 examined by Western blot, *p < 0.05; **p < 0.01; **p < 0.01, compared with group without treatment of Tan IIA. The data were from at least three experiments.

The above results confirmed the effect of Tan IIA on EMT. We then investigated the role of Tan IIA on CRC metastasis *in vitro*. Transwell assay showed that Tan IIA inhibited the migration of colorectal cancer cells in a concentration-dependent manner compared with control group **(** Figures 3A–D **)**. Similar to the results of Transwell assay, wound-healing assay showed that the migratory ability of CRC cells was suppressed after cells were treated with Tan IIA for 48 h **(** Figures 3E–H **)**. Extracellular matrix (ECM) is the physical barrier in the process of tumor metastasis. The change of composition or structure of ECM is closely associated with invasion and metastasis of tumor cells. It was found that, the expression of MMP-2 and MMP-9 decreased after cells were treated with Tan IIA **(** Figures 3I–L **)**. This result was in consistent with a previous analysis, which reported that abnormal expression of matrix metalloproteinases (MMPs) especially MMP-2 and MMP-9 accounts for degradation of ECM (González-Arriaga et al., 2012; Shrestha et al., 2016).

### Tan IIA Inhibited Metastasis of Colorectal Cancer Cells via β-Arrestin1-Mediated β-Catenin Signaling Pathway

**FIGURE 4:**
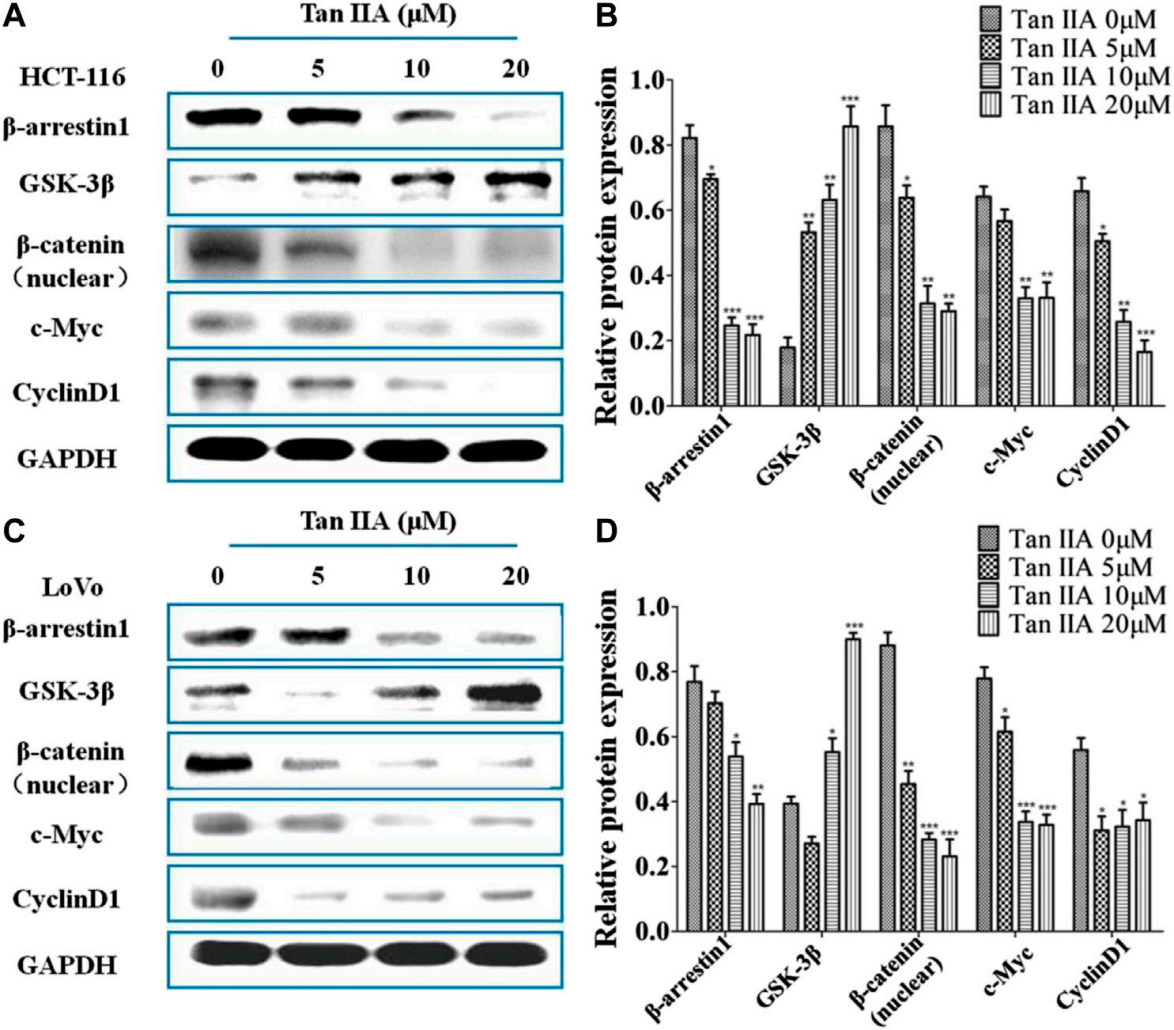
Tan IIA inhibited β-arrestin1-mediated β-catenin signaling pathway **(A,B)** Western blot analysis on the expression of β-arrestin1, GSK-3β, β-catenin, c-Myc, and CyclinD1 in HCT-116 cells with or without Tan IIA treatment, *p < 0.05; **p < 0.01; ***p < 0.001, compared with the group without Tan IIA. The data were from at least three experiments. **(C,D)** Western blot analysis on the level of β-arrestin1, GSK-3β, β-catenin, and downstream genes c-Myc and CyclinD1 in LoVo cells treated with Tan IIA at 0, 5, 10, and 20 μM. *p < 0.05; **p < 0.01; ***p < 0.001, compared with the group without Tan IIA. The data were from at least three experiments.

Inactivation of GSK-3β phosphorylation resulted in nuclear localization of β-catenin, which leads to the activation of GSK/β-catenin signaling pathway and promotes tumor metastasis (Yu et al., 2014). To study whether the anti-metastatic effect of Tan IIA was associated with β-arrestin1/β-catenin signaling pathway in HCT-116 and LoVo cells, GSK-3β, β-catenin, and the downstream genes of β-catenin signaling c-Myc and CyclinD1 were detected by Western blot. It was found that the level of β-arrestin1 was reduced, while the level of GSK-3β was increased when cells were treated with Tan IIA. Further studies showed that Tan IIA inhibited β-catenin translocating into nucleus, and decreased the accumulation of β-catenin in the nucleus, with a corresponding reduction in c-Myc and CyclinD1 **(** Figures 4A–D **)**.

### Tan IIA Inhibited Colorectal Cancer Metastasis via β-Arrestin1/β-Catenin Signaling Pathway *In Vivo*

**FIGURE 5:**
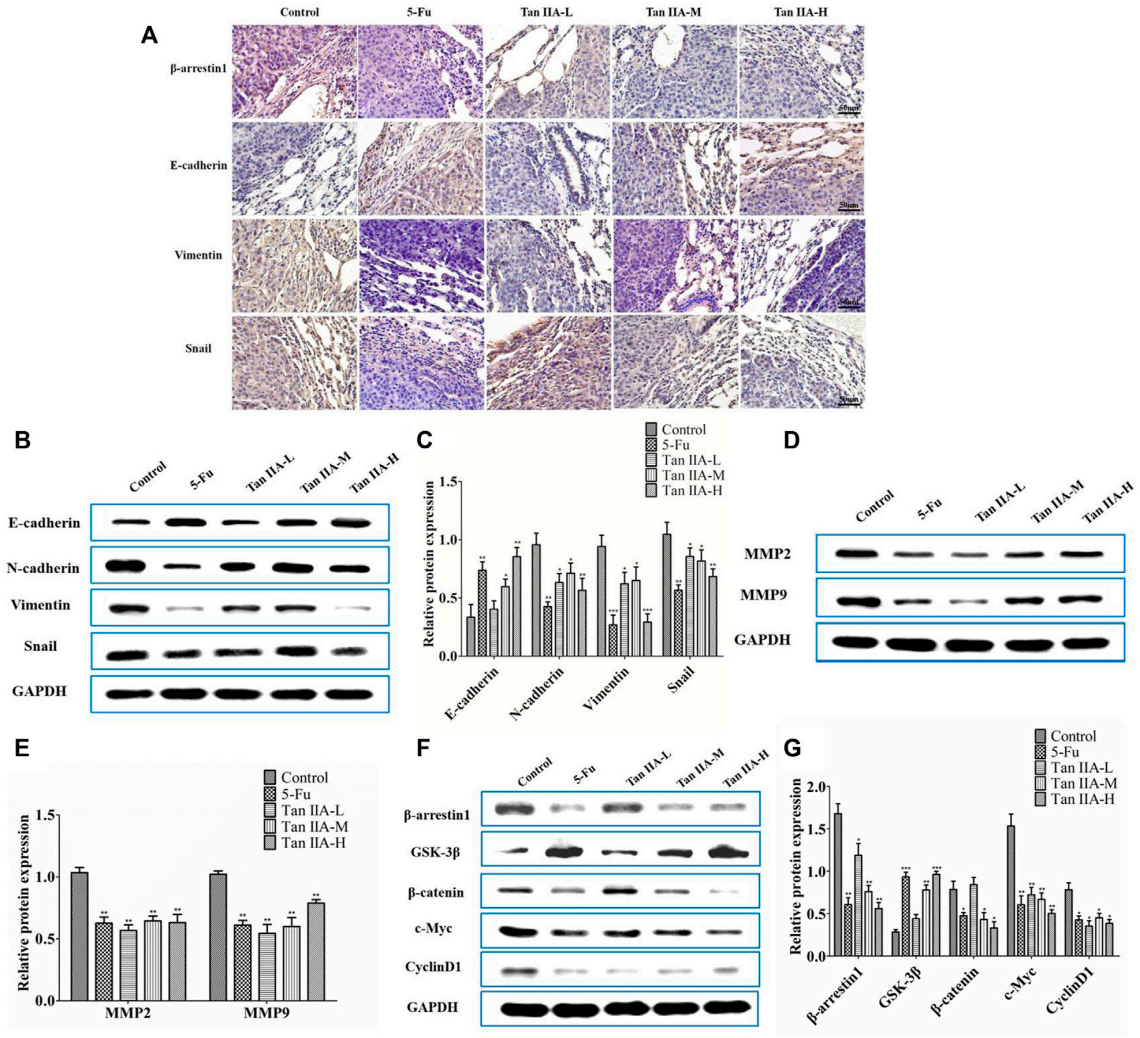
Tan IIA inhibited metastasis of CRC via β-arrestin1/β-catenin signaling pathway *in vivo*. **(A)** Immunohistochemistry on the expression of β-arrestin1, E-cadherin, Vimentin, and Snail in lung tumor tissues. **(B,C)** Western blot on the expression of E-cadherin, N-cadherin, Snail, and Vimentin, *p < 0.05; **p < 0.01; ***p < 0.001, compared with control group. The experiment was performed three times with similar results. **(D,E)** Western blot on the levels of MMP-2 and MMP-9. The data were presented as the mean ± SD from at least three experiments. **p < 0.01, compared with control group. **(F,G)** Western blot on the protein expression of β-arrestin1, GSK-3β, β-catenin, c-Myc, and CyclinD1. *p < 0.05; **p < 0.01; ***p < 0.001, compared with control group. The data were presented from at least three experiments.

The metastasis-related proteins in each group of lung metastases were investigated by immunohistochemistry and Western blot. Immunohistochemical staining showed that Tan IIA decreased the level of β-arrestin1, Vimentin, and Snail protein, while increased expression of E-cadherin protein in a concentration-dependent manner **(** Figure 5A **)**. Consistent with the previous results, lung metastatic tumor treated with Tan IIA had a higher expression of E-cadherin and lower expression of N-cadherin, Vimentin, and Snail compared with those from the tumor without Tan IIA treatment **(** Figures 5B,C **)**. Further detection of metastasis-related proteins found that the levels of MMP-2 and MMP-9 were reduced after Tan IIA treatment **(** Figures 5D,E **)**. The Wnt/β-catenin signaling activity was partly blocked by Tan IIA. The result showed that Tan IIA down-regulated the expression of β-arrestin1, β-catenin and the downstream gene of β-catenin, such as c-Myc and CyclinD1, while up-regulated the protein expression of GSK-3β in a concentration-dependent manner **(** Figures 5F,G **).**

## Discussion

Tan IIA, which is from Salvia miltiorrhiza, has been proved to have coronary artery dilatation, antioxidant and anti-inflammatory effects (Tang et al., 2007; Dong et al., 2009). Recent studies have shown that Tan IIA has anti-cancer effect in various malignant tumors, such as colon cancer, breast cancer, and ovarian cancer (Yu et al., 2014; Bai et al., 2016; Li et al., 2018). Our previous studies demonstrated that Tan IIA is effective against invasion and metastasis of CRC (Sui et al., 2017). EMT is characterized by loss of polarity of epithelial cells and acquisition of mesenchymal properties, which contributes to invasion and distant metastasis of tumor cells (Iwatsuki et al., 2010). When EMT is triggered, the expression of epithelial biomarker E-cadherin and mesenchymal biomarks Vimentin changes (Peinado et al., 2007). In this study, we showed that Tan IIA inhibited lung metastasis of CRC and improved the survival of tumor-bearing mice via suppressing the EMT process. Also, Tan IIA upregulated the expression of E-cadherin and downregulated Vimentin levels through inhibiting the expression of Snail transcription factors in a concentration-dependent manner *in vitro*. EMT-related factors Slug and ZEB1 have been found to play important roles as facilitators of brain metastasis (Nagaishi et al., 2017). Therefore, we have also studied the effect of Tan IIA on Slug and ZEB1, and the data showed that Tan IIA had no significant effect on the expression of Slug and ZEB1 (Supplementary Figure S2). In addition, it was found that the expression of MMP-2 and MMP-9 were decreased after cells were treated with Tan IIA.

A number of studies have found that Wnt/β-catenin is involved in EMT and tumor invasion and metastasis (Duan et al., 2016; Wang et al., 2018; Zhang et al., 2018b). When canonical Wnt signaling pathway is abnormally activated, the function of Axin, APC, GSK-3β, and CK1α complex is greatly restricted, accumulated β-catenin enters into the nucleus from cytoplasm, then the β-catenin/TCF/LEF complex transcriptional activates Snail directly and triggers EMT (Clevers, 2006; Stemmer et al., 2008). β-arrestin1 serves as scaffold proteins participating in multiple signaling pathways (Wang et al., 2019). Our previous study indicated that β-arrestin1 promoted EMT through β-catenin signaling pathway in CRC progression *in vivo* and *in vitro*. The mechanism of β-arrestin1 regulating EMT and metastasis is partly through decreasing the expression of GSK-3β, leading to the accumulation of β-catenin in the cytoplasm and promoting β-catenin to get into the nucleus. In the present study, *in vitro* experimental results demonstrated that Tan IIA inhibited the expression of β-arrestin1 while promoted the expression of GSK-3β, thus blocking β-catenin entry into the nucleus. Then, the expression of c-Myc and CyclinD1 were reduced as a result of the inhibition of β-catenin. Moreover, *in vivo* experiments obtained a similar result of Tan IIA, demonstrated the effects of Tan IIA on EMT, β-catenin signaling pathway and its related proteins.

## Conclusion

**FIGURE 6:**
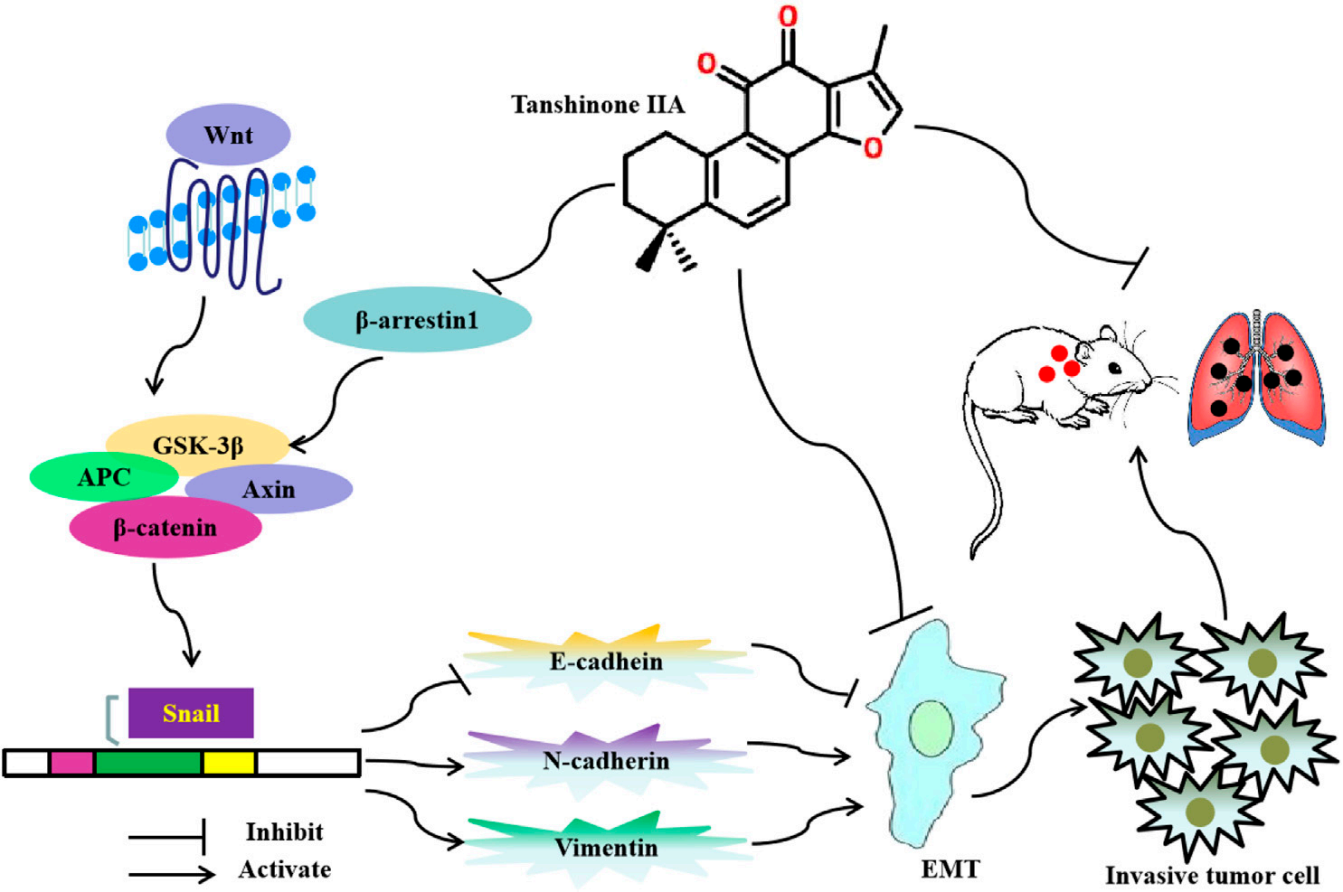
Diagram showing the mechanism of Tanshinone IIA on EMT and metastasis in colorectal cancer.

In summary, this study found that Tan IIA inhibits EMT and metastasis of CRC through repressing β-arrestin1-mediated β-catenin signaling pathway. The potential anti-metastatic mechanism is summarized in Figure 6.

## Data Availability

The raw data supporting the conclusions of this article will be made available by the authors, without undue reservation, to any qualified researcher.
