# Corrigendum: Tanshinone IIA Inhibits Epithelial-to-Mesenchymal Transition Through Hindering β-Arrestin1 Mediated β-Catenin Signaling Pathway in Colorectal Cancer

**DOI:** 10.3389/fphar.2022.929656

**Published:** 2022-07-01

**Authors:** Qing Song, Liu Yang, Zhifen Han, Xinnan Wu, Ruixiao Li, Lihong Zhou, Ningning Liu, Hua Sui, Jianfeng Cai, Yan Wang, Qing Ji, Qi Li

**Affiliations:** ^1^ Department of Medical Oncology and Cancer Institute of Integrative Medicine, Shuguang Hospital, Shanghai University of Traditional Chinese Medicine, Shanghai, China; ^2^ Academy of Integrative Medicine, Shanghai University of Traditional Chinese Medicine, Shanghai, China; ^3^ Department of Medical Oncology, Suzhou TCM Hospital Affiliated to Nanjing University of Chinese Medicine, Suzhou, China; ^4^ Department of Oncology, Baoshan Branch, Shuguang Hospital Affiliated to Shanghai University of Traditional Chinese Medicine, Shanghai, China; ^5^ Department of Chemistry, University of South Florida, Tampa, FL, United States

**Keywords:** tanshinone IIA, colorectal cancer, epithelial-to-mesenchymal transition, β-arrestin1, β-catenin signaling pathway

In the original article, there was a mistake in Figures 1, 3, 5 as published. In Figure 1A, one set of *in vivo* imaging pictures for Tan IIA-M and Tan IIA-H group were incorrectly used for the representative pictures, accompanying with the corresponding quantitative picture in Figure 1B. In Figure 3A, the picture for Tan IIA (5 μM) group was incorrectly used for the representative picture, accompanying with the corresponding quantitative picture in Figure 3B. In Figure 3E, the picture for Tan IIA (10 μM, 0 h) group was incorrectly used for the representative picture. In Figure 5A, the immunohistochemical picture for Snail (Tan IIA-M group) was incorrectly used for the representative picture. The corrected Figures 1, 3, 5 appear below.

**FIGURE 1:**
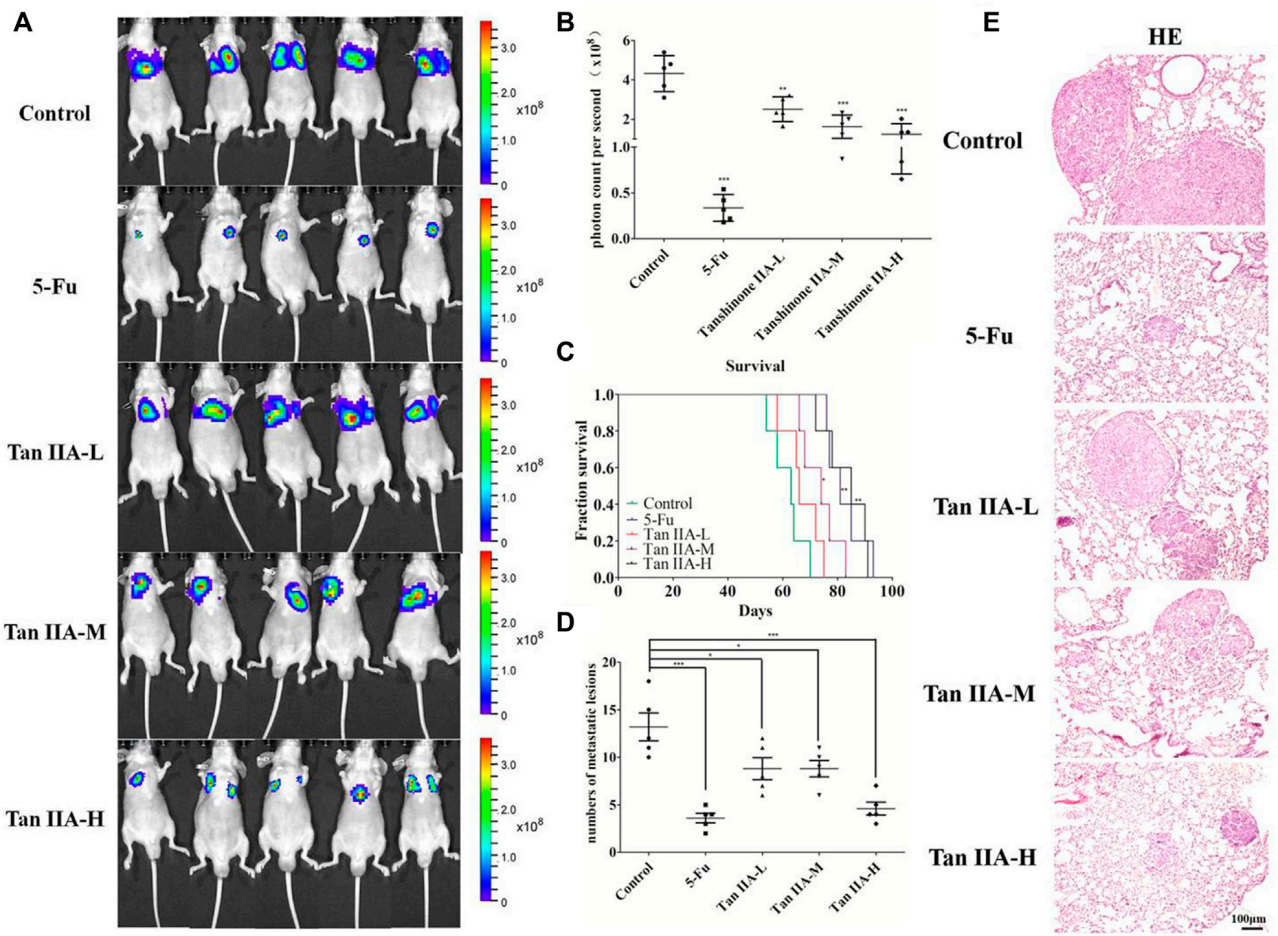
Tan IIA inhibited the metastasis of colorectal cancer *in vivo*. **(A,B)** Each group of mice was injected with HCT-116/luc cells through the tail vein. After treatment with Tan IIA at concentrations of 0.5, 1, and 2 mg/kg for 4 weeks, luciferase imaging data was collected by IVIS Lumina system. ***p < 0.001, compared with control group. **(C)** The survival of tumor-bearing mice were evaluated, *p < 0.05; **p < 0.01; ***p < 0.001, compared with control group. **(D,E)** The lung tumors were excised, hemaoxylin-eosin **(H,E)** staining was performed and the number of metastatic lesions were counted, *p < 0.05; ***p < 0.001, compared with control group.

**FIGURE 3:**
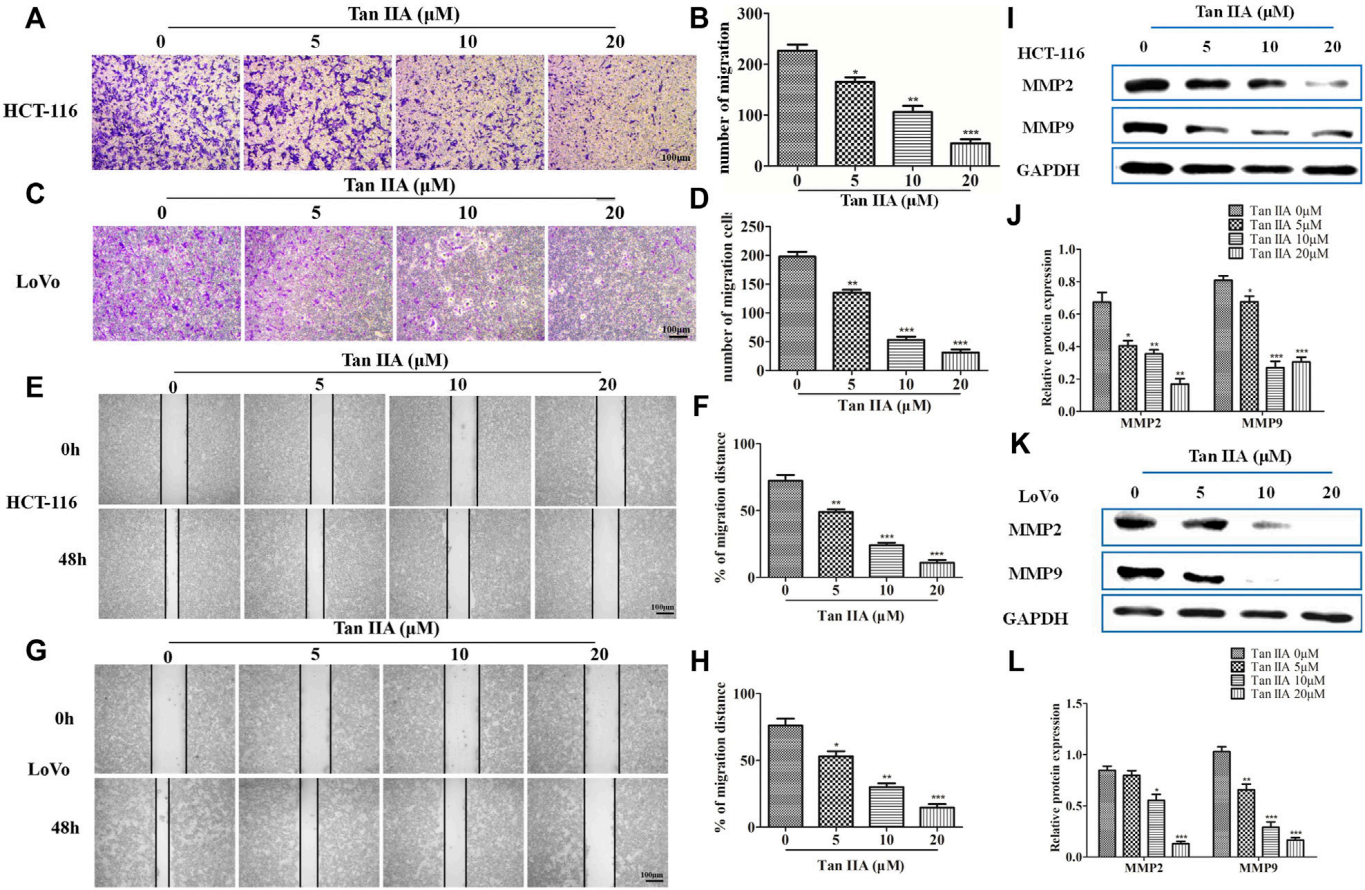
Tan IIA inhibited the migration of CRC cells. **(A–D)** HCT-116 and LoVo cells were treated with different concentration of Tan IIA, and transwell assay was used to detect the migratory cells counted from five random microscopic fields. The experiment was performed three times with similar results. *p < 0.05; **p < 0.01; **p < 0.01, compared with group without treatment of Tan IIA. **(E–H)** HCT-116 and LoVo cells treated with or without Tan IIA for 48 h, the wound-healing assay data were shown. The black lines were used to mark the borders of the scratches, *p < 0.05; **p < 0.01; **p < 0.01, compared with group without treatment of Tan IIA. The data were presented as the mean ± SD from at least three experiments. **(I–L)** The expression of MMP-2 and MMP-9 examined by Western blot, *p < 0.05; **p < 0.01; **p < 0.01, compared with group without treatment of Tan IIA. The data were from at least three experiments.

**FIGURE 5:**
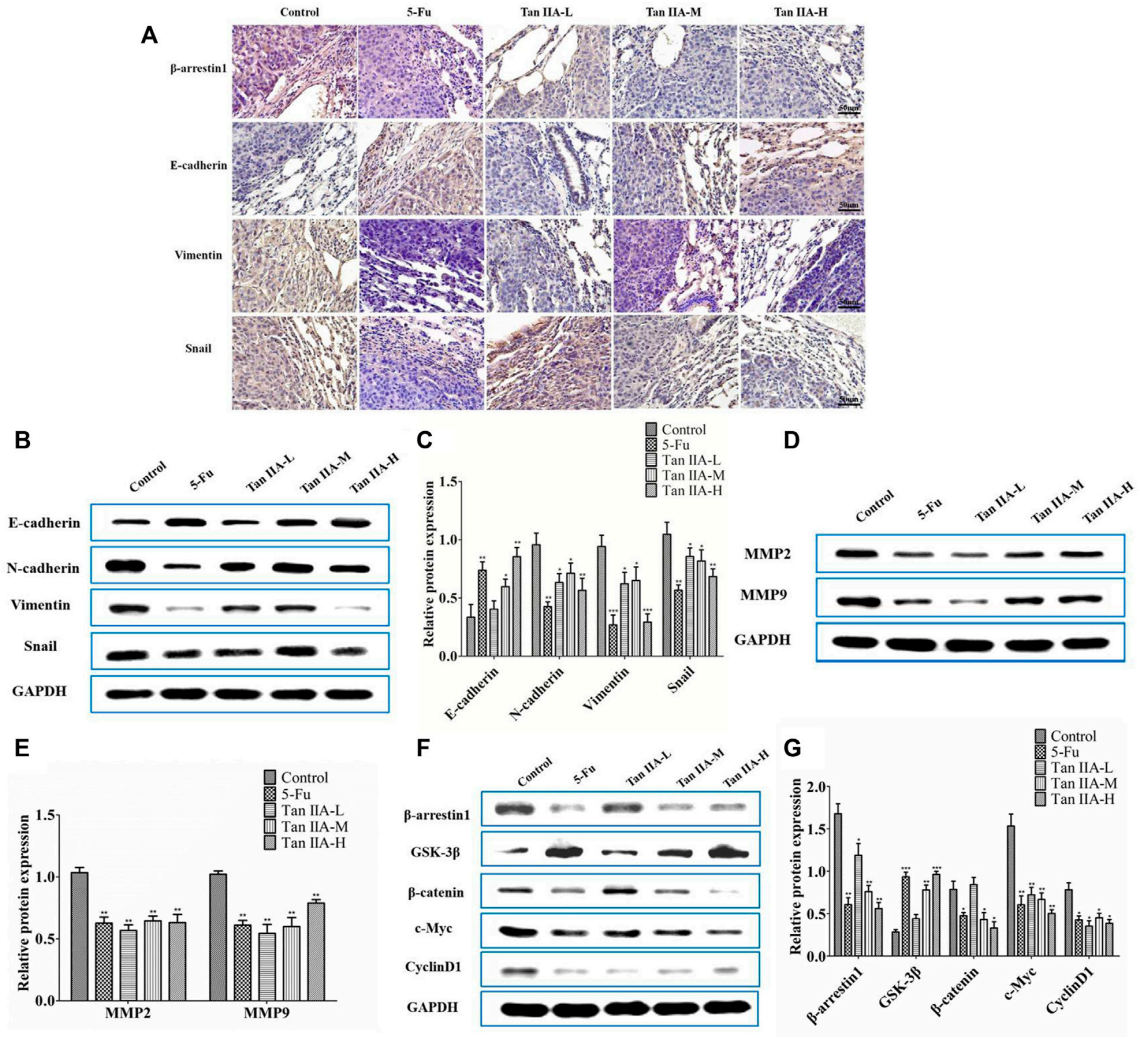
Tan IIA inhibited metastasis of CRC *via* β-arrestin1/β-catenin signaling pathway *in vivo*. **(A)** Immunohistochemistry on the expression of β-arrestin1, E-cadherin, Vimentin, and Snail in lung tumor tissues. **(B,C)** Western blot on the expression of E-cadherin, N-cadherin, Snail, and Vimentin, *p < 0.05; **p < 0.01; ***p < 0.001, compared with control group. The experiment was performed three times with similar results. **(D,E)** Western blot on the levels of MMP-2 and MMP-9. The data were presented as the mean ± SD from at least three experiments. **p < 0.01, compared with control group. **(F,G)** Western blot on the protein expression of β-arrestin1, GSK3β, β-catenin, c-Myc, and CyclinD1. *p < 0.05; **p < 0.01; ***p < 0.001, compared with control group. The data were presented from at least three experiments.

The authors apologize for this error and state that this does not change the scientific conclusions of the article in any way. The original article has been updated.

