# Supplementary material for: Tanshinone IIA Inhibits Epithelial-to-Mesenchymal Transition Through Hindering β-Arrestin1 Mediated β-Catenin Signaling Pathway in Colorectal Cancer
Source: Front Pharmacol. 2020 Oct 29;11:586616. doi: 10.3389/fphar.2020.586616 (PMC7658606; doi:10.3389/fphar.2020.586616)
Supplement: Supplementary file 1 [file Image2.pdf]

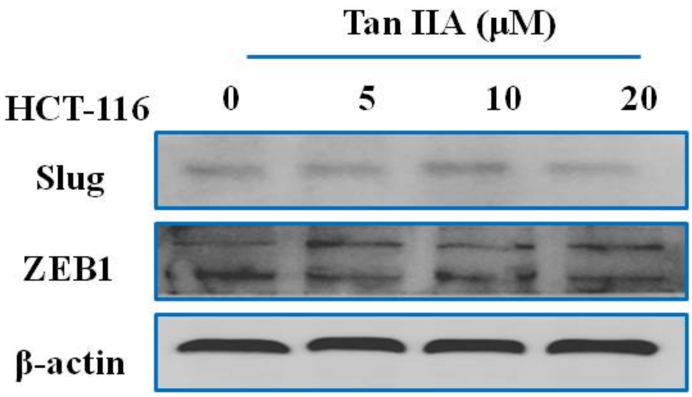

Supplementary Figure 2 Effect of Tan IIA on the expression of Slug and ZEB1. HCT-116 cells were treated with Tan IIA at 0 μM, 5 μM, 10 μM, and 20 μM, Western blot was used to detect the expression of Slug and ZEB1.
