# Supplementary material for: Tanshinone IIA Inhibits Epithelial-to-Mesenchymal Transition Through Hindering β-Arrestin1 Mediated β-Catenin Signaling Pathway in Colorectal Cancer
Source: Front Pharmacol. 2020 Oct 29;11:586616. doi: 10.3389/fphar.2020.586616 (PMC7658606; doi:10.3389/fphar.2020.586616)
Supplement: Supplementary file 2 [file Image1.pdf]

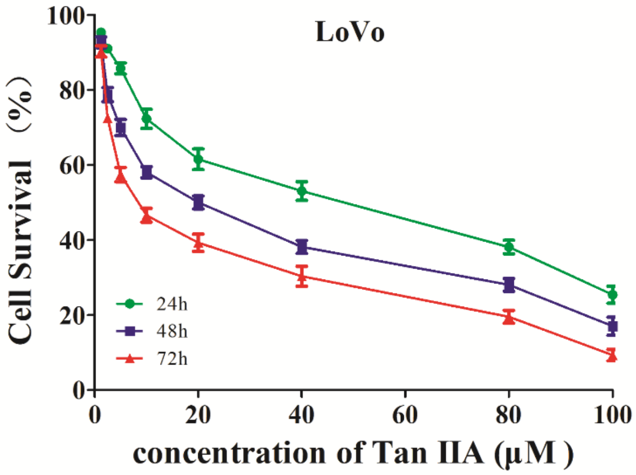

Supplementary Figure 1 Effect of f Tan IIA on the proliferation of LoVo cells. CCK-8 assay on the cellular inhibition of Tan IIA (0, 1.25, 2.5, 5, 10, 20, 40, 80, and 100 μM) in LoVo cells for 24, 48, and 72 h. The experiment was performed three times with similar results.
